# Breast density and breast cancer-specific survival by detection mode

**DOI:** 10.1186/s12885-018-4316-7

**Published:** 2018-04-05

**Authors:** Daniëlle van der Waal, André L. M. Verbeek, Mireille J. M. Broeders

**Affiliations:** 1 Radboud Institute for Health Sciences (Department for Health Evidence, Mailbox 133), Radboud university medical center, PO Box 9101, Nijmegen, 6500 HB The Netherlands; 2 Dutch Expert Centre for Screening, PO Box 6873, Nijmegen, 6503 GJ The Netherlands

**Keywords:** Breast cancer survival, Screening, Breast density, Detection mode

## Abstract

**Background:**

Breast density is known to affect breast cancer risk and screening sensitivity, but it may also be associated with breast cancer survival. The interpretation of results from previous studies on breast density and survival is complicated by the association between detection mode and survival. Here, we studied the effect of breast density on breast cancer-specific survival for different detection modes (screen-detected, interval ≤ 24 or > 24 months, non-participant).

**Methods:**

Data from the Nijmegen (Dutch) breast cancer screening programme were used. Women diagnosed with invasive breast cancer between 1975 and 2011 were included. Breast density was assessed visually, based on a dichotomized Wolfe scale: ‘fatty breasts’ (≤25%) and ‘dense breasts’ (> 25%). Cox proportional hazard regression was used to obtain hazard ratios (HR).

**Results:**

We identified 2742 eligible women, with a breast pattern available for 2233 women. A diagnosis of interval cancer (HR 2.06, 95% CI 1.62–2.61) led to a significantly increased risk of breast cancer death compared with screen-detected cancer. No significant cause-specific survival difference between women with dense and fatty breasts was observed (HR 0.94, 95% CI 0.77–1.15). The hazard was only higher for women with dense breasts among interval cancers ≤24 m (HR 1.07, 95% CI 0.74–1.56). The hazard appeared to be lower for women with dense breasts than for women with fatty breasts among screen-detected (HR 0.77, 95% CI 0.53–1.11) and interval cancers > 24 m (HR 0.80, 95% CI 0.53–1.20). None of the effects were statistically significant.

**Conclusions:**

Detection mode is strongly associated with breast cancer death. No clear association is apparent between breast density and breast cancer death, regardless of detection mode.

## Background

Fibroglandular breast tissue is radiodense and appears as white areas on a mammogram. High breast density, i.e., a high proportion of dense tissue, has been associated with an increased breast cancer risk. The relative risk is estimated at 4 to 6 when women with more than 75% dense tissue are compared with women with less than 25% dense tissue [1]. In addition, dense breast tissue masks tumours on mammograms: tumour detection is decreased when breast density is high [2]. This results in a lower programme sensitivity in breast cancer screening in women with dense breasts.

It is unclear to what degree breast density is associated with breast cancer survival. Two different hypotheses may be suggested. High breast density delays cancer detection due to the masking effect [2]. Tumours occurring in dense breasts may thus on average have progressed to a higher stage, with worse prognostic characteristics, at diagnosis. This would affect breast cancer survival. Some studies have indeed observed an association between high breast density and indicators of tumour progression, although results are inconclusive [3–7]. Examples include larger tumour size and lymph node involvement.

The second hypothesis is based on an association with tumour aggressiveness. The etiological pathway through which breast density increases breast cancer risk is unknown. Breast tumours often originate from epithelial tissue. A higher number of epithelial cells and a potentially increased proliferation in dense tissue may thus explain the association, although an important role for the stromal tissue has also been suggested [8, 9]. Whether the natural history of these tumours differs from tumours arising in fatty breasts has, however, not been determined. Some breast cancer risk factors (e.g., body-mass index [BMI] and several reproductive factors) are known to affect the risk of tumour subtypes differently, which may translate into a different disease prognosis and breast cancer survival [10–12]. The evidence on breast density and its possible involvement in pathways that lead to more aggressive tumours is still ambiguous [3–7, 13–18]. A higher tumour aggressiveness, as indicated by tumour markers including hormone receptor status, also results in higher stage at diagnosis and poorer breast cancer survival.

The difficulty with survival analyses is that they may be affected by lead time bias when the study is conducted in a screening setting. Lead time is the time by which a breast cancer diagnosis is advanced due to early detection [19]. If survival time is measured as the time since diagnosis, the advanced diagnosis would increase survival time, even without an actual effect on breast cancer survival. Since the distribution of screen-detected and interval tumours is expected to be different in women with high breast density compared with women with low breast density, this may affect survival estimates as well. To gain more insight into these different effects, the association between density and breast cancer survival could be studied separately for each detection mode, in addition to studying overall breast cancer survival.

Previous studies have addressed the effect of breast density on breast cancer survival or breast cancer mortality, but the results have been inconclusive [20–28]. The studies by Chiu et al. and Olsson et al. appeared to suggest an increased mortality in dense breasts, whereas other studies found no significant difference or even observed a potential survival benefit. Detection mode (i.e., screen-detected cancer, interval cancer, or cancer in non-participants) was not taken into account in most of these studies, apart from Olsson [21] and Eriksson [24], making it difficult to compare results and explain the observed differences.

In this study, we addressed the effect of breast density pattern on breast cancer-specific survival. Breast cancer-specific survival was studied for different modes of detection, in order to remove or reduce the potential role of lead and length time bias in our associations.

## Methods

### Population

The study was based on data from the Nijmegen (the Netherlands) breast cancer screening population. A pilot screening programme started here in 1975, with women 35 years or older being invited [29]. In 1989, a centralized nationwide programme was introduced in the Netherlands. This programme was aimed at women 50 to 69 years old. The screening age in Nijmegen was adapted accordingly. Since 1998, Dutch women aged 70 to 74 years also receive a screening invitation. The current programme invites women 50 to 74 years to mammographic screening every 2 years. Every mammogram is read independently by two certified screening radiologists. Women included in our study received at least one screening invitation before their diagnosis. All women consented to the use of their anonymous data for scientific research.

### Breast cancer patients

Women diagnosed with invasive breast cancer between 1975 and 2011, both via screening and outside the screening programme, were included in our study. Women were excluded if it was unclear if the tumour truly had an invasive histology (*n* = 37). The diagnosis date was defined as the date at which clinical mammography was performed (*n* = 2655). When this date was unavailable, the date was set at 1 month prior to surgery (*n* = 77) or the date was obtained from the pathology report (*n* = 10). Women were excluded from the study when these dates were missing (*n* = 11). Information on vital status and cause of death were obtained via linkage with municipality records.

Women were followed from their diagnosis date until date of death, date of moving out of the area, or the end of study follow-up (December 31st, 2011). Follow-up was not always complete: it was unclear if some women were still alive, but no report of relocation or death was available (*n* = 652). The end date was then set at the year after the last screening invitation for a sensitivity analysis. We studied the effect of this censoring on the estimates.

Mode of detection was categorised into screen-detected, interval, and non-participant cancer. Screen-detected cancer was defined as all diagnoses after a positive screening exam. Interval cancer was further divided into tumours occurring within 24 months (≤24 m) and more than 24 months (> 24 m) after a negative screening exam. The category ‘non-participant cancer’ consists of cancer diagnoses in women who never participated in breast cancer screening.

### Breast density

Screening is based on two-view mammography at first examination: mediolateral oblique (MLO) and craniocaudal (CC). In the time period that we studied, subsequent exams consisted of one view (MLO), with the CC view being used on indication. Digital mammography was introduced in the Nijmegen screening programme in 2007. Density was, however, only assessed on film-screen mammograms.

Breast density was assessed visually by a trained research assistant. These assessments were based on all available mammography views. A quantified version of the Wolfe breast pattern scale was used, which consisted of the following categories: (1) < 5%, (2) 5–25%, (3) 26–75%, and (4) > 75% breast density. This scale was dichotomized for all analyses into ‘fatty breasts’ (≤25%) and ‘dense breasts’ (> 25%). A previous study has shown that there is a strong correlation between the Wolfe and the BI-RADS scale [30]. Breast density measurements were based on the last screening round before diagnosis (*n* = 1515). If this was not available, breast density was based on the diagnostic mammogram (*n* = 612) or the round preceding the last screening round (*n* = 106). Women were excluded from the analyses on breast density when no breast pattern was available in our dataset (*n* = 509).

### Statistical analyses

Cox proportional hazard regression was used to assess the effect of breast density on breast cancer-specific survival. We thus report cause-specific survival estimates, rather than e..g. overall survival or disease-free survival. Breast density was included as a determinant in the analyses. This resulted in hazard ratios (HR) and corresponding 95% confidence intervals (CI). The time scale was from time of diagnosis to date of breast cancer death. Analyses were stratified by mode of detection. All analyses were corrected for age at diagnosis.

Kaplan-Meier curves were examined and time-dependent covariates were included in the regression model to test the proportional hazards assumptions. All tests were two-sided, with a *p*-value < 0.05 being considered statistically significant. The analyses were performed with SPSS 22.0.

## Results

We identified 2742 eligible women with an invasive tumour, with a breast pattern available for 2233 women. The age at diagnosis ranged from 38 to 97 years. Most women had a fatty breast pattern (*n* = 1456, 65.2%). There were 536 breast cancer deaths in this population. Breast density was known for 479 breast cancer deaths, of whom 173 (36.1%) had dense breasts. There were 149 breast cancer deaths among the 1257 screen-detected cases, 129 among the 557 interval cases ≤24 m, 154 among the 604 interval cases > 24 m, and 104 among the 324 non-participant cases.

### Breast cancer-specific survival by detection mode

The age-adjusted effect of detection mode on breast cancer-specific survival is presented in Table 1. Screen-detected cancer was used as the reference category. A diagnosis of interval cancer ≤24 m (age-adjusted HR 2.06, 95% CI 1.62–2.61), interval cancer > 24 m (age-adjusted HR 3.00, 95% CI 2.37–3.79), and non-participant cancer (age-adjusted HR 4.18, 95% CI 3.25–5.39) led to a significantly poorer breast cancer-specific survival compared with screen-detected cancer.

**Table 1:** Breast cancer-specific survival by detection mode

	Person years	N total^a^	N breast cancer deaths (%)	HR adjusted for age (95% CI)	*P*-value
Screen-detected cancer	13,183	1257	149 (11.9)	1.00 (Ref.)	
Interval cancer ≤24 months	5627	557	129 (23.2)	2.06 (1.62–2.61)	< 0.001
Interval cancer > 24 months	4299	604	154 (25.5)	3.00 (2.37–3.79)	< 0.001
Non-participant cancer	2097	324	104 (32.1)	4.18 (3.25–5.39)	< 0.001

^a^ Follow-up time of 11 participants was censored before the first death occurred in the stratum

### Breast cancer-specific survival by breast density

As shown in Table 2, there was no significant difference in breast cancer-specific survival between dense and fatty breasts when all detection modes were combined (age-adjusted HR 0.94, 95% CI 0.77–1.15). The hazard was only slightly higher for women with dense breasts among interval tumours ≤24 m (age-adjusted HR 1.07, 95% CI 0.74–1.56). The hazard appeared to be lower in women with dense breasts than fatty breasts among screen-detected (age-adjusted HR 0.77, 95% CI 0.53–1.11) and interval tumours > 24 m (age-adjusted HR 0.80, 95% CI 0.53–1.20). When screen-detected and interval tumours ≤24 m were combined, there appeared to be no difference in hazard (age-adjusted HR 1.01, 95% CI 0.78–1.31). None of the effects were, however, statistically significant. Adjustment of the overall effect for age and mode of detection resulted in a HR of 0.84 (95% CI 0.68–1.03), possibly suggesting an inverse association between high breast density and breast cancer-specific survival.

**Table 2:** Breast cancer-specific survival by breast density and mode of detection

	Person years	N total^a^	N breast cancer deaths (%)	HR adjusted for age (95% CI)^b^	*P*-value	HR adjusted for age and mode of detection (95% CI)^b^	*P*-value
Overall							
Dense	9418	777	173 (22.3)	0.94 (0.77–1.15)	0.58	0.84 (0.68–1.03)	0.09
Fatty	14,495	1456	306 (21.0)	1.00 (Ref.)		1.00 (Ref.)	
Screen-detected + Interval cancer ≤ 24 m							
Dense	7424	595	115 (19.3)	1.01 (0.78–1.31)	0.91		
Fatty	10,995	995	162 (16.3)	1.00 (Ref.)			
Screen-detected cancer							
Dense	4329	340	44 (12.9)	0.77 (0.53–1.11)	0.16		
Fatty	8589	760	105 (13.8)	1.00 (Ref.)			
Interval cancer ≤ 24 m							
Dense	3095	255	71 (27.8)	1.07 (0.74–1.56)	0.72		
Fatty	2406	235	57 (24.3)	1.00 (Ref.)			
Interval cancer > 24 m							
Dense	1433	133	41 (30.8)	0.80 (0.53–1.20)	0.29		
Fatty	2334	290	86 (29.7)	1.00 (Ref.)			
Non-participant cancer							
Dense	562	49	17 (34.7)	0.72 (0.40–1.31)	0.29		
Fatty	1166	171	58 (33.9)	1.00 (Ref.)			

^a^ Follow-up time of several participants was censored before the first death occurred in the stratum. Overall: *n* = 4; Screen-detected+Interval cancer ≤24 m: *n* = 8; Screen-detected cancer: *n* = 5; Interval cancer ≤24 m: n = 4; Interval cancer > 24 m: *n* = 1; Non-participant cancer: *n* = 1

Additional adjustment for year of diagnosis did not change the estimates (data not shown). Furthermore, sensitivity analyses showed that censoring the follow-up after last invitation in women with apparent loss to follow-up had no effect on the estimates (data not shown).

## Discussion

There was no clear overall association between breast density and breast cancer-specific survival. A high breast density might lead to missed detection and thus a later stage at diagnosis, when tumours are harder to treat (i.e. a true worsening of survival). Since screen-detected tumours are relatively less frequent in women with dense breasts due to missed detection, however, survival estimates are less affected by lead and length time bias in women with high breast density. These biases make survival associated with low breast density appear better, thus further increasing the difference in breast cancer-specific survival. By adjusting for mode of detection, we attempted to remove lead and length time bias from our association. This adjustment, however, also removed that part of the true association between breast density and breast cancer-specific survival due to missed detection’s influence on later stage at diagnosis and subsequent worse survival. The possible inverse association between breast density and breast cancer-specific survival after adjustment would thus have to be attributed to other factors.

Breast cancer-specific survival in interval cancer is always expected to be worse than in screen-detected cancer [31]. The difference in breast cancer-specific survival (HR 2.06, 95% CI 1.62–2.61) may partly be explained by lead or length time bias, resulting in an artificial increase in survival time due to the advanced diagnosis in screen-detected cancer or the detection of slow-growing tumours. The remaining breast cancer-specific survival difference would be explained by a better prognosis as a result of early detection. Detection mode was previously shown to give important prognostic information, even after adjustment for well-known prognostic characteristics [32].

The distribution of interval and screen-detected tumours is expected to differ between women with high and low breast density. As a result of the masking effect of dense tissue and subsequent missed detection, the risk of interval tumours is higher [20, 33–36]. Tumours are thus detected at a later stage, when they are harder to treat. Newer mammography systems are expected to decrease the masking effect. Although mammographic technology has greatly evolved between 1975 and 2011, adjustment for year of diagnosis did not appear to alter the estimates (results not shown). This suggests that the association, or rather lack of association, is consistent over time.

After adjusting the overall estimates for detection mode, women with dense breasts appeared to have a survival advantage compared to women with fatty breasts. This potential inverse association between breast density would have to be explained by other factors than missed detection, e.g. an inverse association with tumour aggressiveness, but we have to be cautious with the interpretation of these results. There are several studies on the association between breast density and prognostic tumour characteristics [3–7, 13–18]. Results on associations with tumour markers of aggressiveness, e.g. hormone receptor status, appear to be inconclusive. Some studies observed associations with tumour markers of progression, e.g. large tumour size and lymph node involvement, which may be a consequence of late detection. Most studies were not able to differentiate between screen-detected and interval tumours, thus limiting conclusions that may be drawn on the contribution of late detection to these associations.

Several previous studies have addressed the association between breast density and breast cancer mortality or breast cancer-specific survival, with large variation in design, outcomes, and conclusions (Table 3) [20–28]. Olsson et al. was the only one to stratify by mode of detection [21]. Dense breasts appeared to be associated with an increased risk of breast cancer death in symptomatic and non-symptomatic tumours. Gierach et al. adjusted for mode of detection in the fully adjusted model, but they did not present the stratified results [25]. They found no significant difference in breast cancer-specific survival between dense and fatty breasts. This was true for most other studies as well. Olsen et al. found a higher mortality for dense breasts, but case fatality appeared to be lower [20]. Six studies presented estimates that were adjusted for indicators of tumour progression and/or type of treatment. These adjustments are likely to remove (a large part of) the negative effect of missed detection on breast cancer-specific survival as well. Assuming that there is no biological association with tumour aggressiveness, the adjusted estimates are expected to show no effect. Olsson et al. and Chiu et al., however, found the opposite: the already positive hazard ratios further increased after these adjustments [21, 27]. Maskarinec et al. and Zhang et al. only presented adjusted estimates, which suggested no effect [26, 28]. This was in line with the findings from Gierach and colleagues [25].

**Table 3:** Overview of studies on association between breast density and breast cancer-specific survival

Study	Country	Setting	Study design	Breast cancer deaths (N)	Density measurement	Outcome (univariable or minimally adjusted)	Outcome (multivariable)
Olsen [20]	Denmark	Screening interval 2y, age 50-69y, 1991–2001	Cohort. Inclusion of interval and screen-detected cancer deaths.	Fatty: 37 Mixed/dense: 53	Dichotomized measure with Fatty breasts and Mixed/Dense breasts	*Dense* vs. *Fatty* Case fatality RR 0.53 (0.34–0.82)^a^, Mortality: RR 1.78 (95%CI 1.17–2.72)	
Olsson [21]	Sweden	Screening interval 18 to 24 m, age 50-69y, 1991–1996	Cohort. Inclusion of non-symptomatic and symptomatic cancer deaths.	Fatty: 7 Moderate: 37 Dense: 32	Modification of the BI-RADS classification with Fatty, Moderate, and Dense breasts	*Dense* vs. *Fatty* All: HR 1.92 (0.85–4.35) Non-symptomatic: HR 1.47 (0.41–5.27) Symptomatic: HR 2.33 (0.80–6.80)	*Dense* vs. *Fatty* All: HR 2.63 (1.10–6.30)^b^ Non-symptomatic: HR 2.04 (0.49–8.49) Symptomatic: HR 3.40 (1.06–10.90)
Porter [22]	England	Screening interval 3y, age 50-64y, since 1987	Cohort. Inclusion of interval and screen-detected cancer deaths.	BI-RADS 1: 186 BI-RADS 2: 126 BI-RADS 3: 373 BI-RADS 4: 74	BI-RADS breast density	*Dense* vs. *Fatty* No significant difference all cancer deaths (*p* = 0.12), screen-detected (*p* = 0.75), or interval (*p* = 0.82)	
van Gils [23]	The Netherlands	Screening interval 2y, age 50-69y, 1977–1994	Cohort. Inclusion of screen-detected cancer deaths.	26	Visual assessment fatty (≤ 25%) and dense (> 25%)	*Dense* vs. *Fatty* 10-year survival dense 73% vs. fatty 83% (survival *p* = 0.07) in first rounds, later rounds 10-year survival dense 89% vs. fatty 96% (no significant difference, *p* = 0.48)	
Eriksson [24]	Sweden	Screening interval 24 m, age 50-74y, 1993–1995	Cohort. Inclusion of interval and screen-detected cancer deaths.	Dense: 345 Nondense: 1055	Cumulus dichotomized into nondense (< 25%) and dense (≥ 25%)	*Interval* vs. *Screen-detected* All: HR 3.50 (2.25–5.44)^c^ Dense: HR 3.00 (1.26–7.17) Nondense: HR 3.62 (2.17–6.06)	*Interval* vs. *Screen-detected* All: HR 1.69 (1.03–2.76)^d^ Dense: HR 1.26 (0.47–3.38) Nondense: HR 1.76 (1.01–3.09)
Gierach [25]	US	Age > 30y, 1996–2005	Cohort. Inclusion of screen-detected, interval, other screen-detected, clinically detected, and other cancer deaths.	BI-RADS 1: 72 BI-RADS 2: 346 BI-RADS 3: 387 BI-RADS 4: 84	BI-RADS breast density	*Extremely dense* vs. *Scattered* All: HR 0.83 (0.65–1.07)^e^	*Extremely dense* vs. *Scattered* All: HR 0.92 (0.71–1.19)^f^
Maskarinec [26]	US	1993–1996	Case-control.	Low density: 12 High density: 15	Cumulus dichotomized into low (< 35%) and high (> 35%)		*High* vs. *Low percent density* All: HR 0.83 (0.36–1.92)^g^
Chiu [27]	Sweden	Age 45-59y, 1977–2004	Cohort. Mammographically detected, interval cancer, and refuser cases.	Dense: 28 Nondense: 99	Tabár dichotomized into dense (IV and V) and nondense (I-III)	*Dense* vs. *Fatty* All: HR 1.41 (0.92–2.14)	*Dense* vs. *Fatty* All: HR 1.75 (0.99–3.10)^h^
Zhang [28]	US	1994–2008	Cohort.		BI-RADS breast density dichotomized into dense (3 and 4) and fatty (1 and 2)		*Dense* vs. *Fatty* All: HR 0.91 (*p*-value = 0.1242)^i^

In brackets behind the HRs are the 95% confidence intervals

^a^ Case fatality was based on breast cancer death, including all deaths was 0.60. This involves age-adjusted rate ratios

^b^ Adjusted for age at diagnosis, tumour size, axillary lymph node involvement, Nottingham grade, oestrogen receptor, progesterone receptor, diagnosis year, HRT use, BMI, and mode of detection (if applicable)

^c^ Adjusted for age, BMI, and hormone replacement therapy (HRT)

^d^ Adjusted for age, BMI, HRT, and lymph node metastases

^e^ Adjusted for registry site, age at diagnosis, and diagnosis year

^f^ Adjusted for registry site, age at diagnosis, diagnosis year, BMI, mode of detection, surgery/radiation, chemotherapy, and income

^g^ Adjusted for age at diagnosis, ethnicity, obesity, disease stage, and radiation treatment

^h^ Adjusted for age, tumour size, node status, grade, and BMI

^i^ Adjusted for age, ethnicity, and tumour stage

Stratification by detection mode removes, or at least reduces, the effects of missed detection, lead time bias, and length time bias. In the resulting groups (i.e. screen-detected and interval cancer), there are still other effects that need to be considered. A substantial number of interval tumours in the first year after screening in women with high density may actually be slow-growing tumours that were masked during screening (i.e. false-negative results). The first-year interval tumours in women with a low breast density, on the other hand, could be more aggressive fast-growing tumours. Delayed detection due to high breast density could also still have some influence on observed breast cancer-specific survival in symptomatic cases. Furthermore, excessive opportunistic screening provides a challenge in differentiating between symptomatic and non-symptomatic tumours. Opportunistic screening refers to screening activities that take place outside the organised screening programme. For example, asymptomatic women may ask their GP for a referral to the hospital rather than or even in addition to attending the regular screening programme. These women are classified as non-participants, despite participating in ‘opportunistic screening’. Previous research has shown that opportunistic screening occurs frequently [37], and this may have also affected our estimates for the non-participants.

Our study had some limitations. We used a qualitative method to measure breast density, which may have led to non-differential misclassification. Breast pattern was also missing in a relatively large group. This was mainly the result of the introduction of digital mammography. Digital mammography was introduced in the clinic several years before it was introduced in the Dutch screening programme. Breast density pattern was therefore more often missing for ‘interval tumours > 24 m’ and ‘non-participant’ tumours than for ‘screen-detected’ or ‘interval ≤ 24 m’ tumours. It seems unlikely, however, that the missing data is associated with breast density status or breast cancer death. Another limitation is the lack of potential confounder data. We only have accurate and complete information on age at diagnosis and year of diagnosis. We did, however, stratify by mode of detection, which can also be seen as a proxy method for dealing with lead and length time bias. Finally, a certain degree of informative censoring may have potentially affected our findings.

## Conclusions

Detection mode undoubtedly has a strong association with breast cancer death, but there is no clear association between breast density and breast cancer death. This is regardless of detection mode. Breast density is known to affect screening performance and screening decreases the risk of breast cancer death. Further research is still, however, needed on the interplay between breast density, detection mode, and breast cancer-specific survival. Disentangling the different components that influence the observed association between breast density and breast cancer survival will provide new insights into the potential role of breast density in both tumour biology and early detection.

## Abbreviations

CC: Craniocaudal
HR: Hazard ratio
MLO: Mediolateral oblique
